# *De novo* Transcriptome Assembly of Floral Buds of Pineapple and Identification of Differentially Expressed Genes in Response to Ethephon Induction

**DOI:** 10.3389/fpls.2016.00203

**Published:** 2016-02-26

**Authors:** Chuan-He Liu, Chao Fan

**Affiliations:** ^1^Institute of Fruit Tree Research, Guangdong Academy of Agricultural SciencesGuangzhou, China; ^2^Key Laboratory of South Subtropical Fruit Biology, Genetic Resource Utilization Ministry of AgricultureGuangzhou, China

**Keywords:** pineapple [*Ananas comosus* (L.) Merr.], floral induction, ethephon, transcriptome, differentially expressed genes (DEGs)

## Abstract

A remarkable characteristic of pineapple is its ability to undergo floral induction in response to external ethylene stimulation. However, little information is available regarding the molecular mechanism underlying this process. In this study, the differentially expressed genes (DEGs) in plants exposed to 1.80 mL·L^−1^ (T1) or 2.40 mL·L^−1^ ethephon (T2) compared with Ct plants (control, cleaning water) were identified using RNA-seq and gene expression profiling. Illumina sequencing generated 65,825,224 high-quality reads that were assembled into 129,594 unigenes with an average sequence length of 1173 bp. Of these unigenes, 24,775 were assigned to specific KEGG pathways, of which metabolic pathways and biosynthesis of secondary metabolites were the most highly represented. Gene Ontology (GO) analysis of the annotated unigenes revealed that the majority were involved in metabolic and cellular processes, cell and cell part, catalytic activity and binding. Gene expression profiling analysis revealed 3788, 3062, and 758 DEGs in the comparisons of T1 with Ct, T2 with Ct, and T2 with T1, respectively. GO analysis indicated that these DEGs were predominantly annotated to metabolic and cellular processes, cell and cell part, catalytic activity, and binding. KEGG pathway analysis revealed the enrichment of several important pathways among the DEGs, including metabolic pathways, biosynthesis of secondary metabolites and plant hormone signal transduction. Thirteen DEGs were identified as candidate genes associated with the process of floral induction by ethephon, including three *ERF*-like genes, one *ETR*-like gene, one *LTI*-like gene, one *FT*-like gene, one *VRN1*-like gene, three *FRI*-like genes, one *AP1*-like gene, one *CAL*-like gene, and one *AG*-like gene. qPCR analysis indicated that the changes in the expression of these 13 candidate genes were consistent with the alterations in the corresponding RPKM values, confirming the accuracy and credibility of the RNA-seq and gene expression profiling results. Ethephon-mediated induction likely mimics the process of vernalization in the floral transition in pineapple by increasing *LTI, FT*, and *VRN1* expression and promoting the up-regulation of floral meristem identity genes involved in flower development. The candidate genes screened can be used in investigations of the molecular mechanisms of the flowering pathway and of various other biological mechanisms in pineapple.

## Introduction

Pineapple [*Ananas comosus* (L.) Merr.], which is indigenous to Brazil, Argentina and Paraguay, has been introduced worldwide and is the leading edible member of the Bromeliaceae family (Smith and Downs, 1974). It is an economically important tropical herbaceous fruit with attractive sensorial and nutritional characteristics (Coppens d'Eeckenbrugge and Leal, 2003). It is generally consumed fresh as table fruit or in desserts and is used for juice preparation due to its delicate flavor, overall acceptability and nutritional richness in terms of vitamins and minerals (Chauhan et al., 2009).

Flowering is a crucial developmental stage in the plant life cycle. The transition from vegetative growth to flowering is of importance as the first step of sexual reproduction and fruit setting (Bernier et al., 1993). A number of different factors, from environmental to chemical, can trigger flowering. Environmental conditions that promote natural flowering increase the sensitivity of plants to external flowering induction agents. Flowering induction can also be achieved via the use of a range of commercial agents. In pineapple and other bromeliads, it has been proposed that flowering is triggered by a small burst of ethylene production in the meristem in response to environmental cues (Trusov and Botella, 2006). This feature is exploited worldwide by commercial pineapple growers to synchronize flower and fruit development, resulting in a single harvest pass. This synchronization is of crucial importance in pineapple cultivation due to the non-climacteric nature of the fruit (Van de Poel et al., 2009). Ethephon (2-chloroethylphosphonic acid), an ethylene releaser, is universally accepted as the most effective stimulant to induce the flowering of pineapple plants and is used worldwide. Application of ethephon to the central cup induces more homogeneous flowering compared to application of ethylene gas to the entire plant (Van de Poel et al., 2009). With the use of ethephon for flower induction, pineapple can be fruited and harvested multiple times per year (Liu and Liu, 2014).

For annual plants, such as *Arabidopsis thaliana*, proper determination of the flowering time is critical for plant success because the switch from vegetative to reproductive development is irreversible (Klepikova et al., 2015). Consequently, the transition to flowering is under strict genetic and environmental control (Casal et al., 2004), with induction of floral initiation by both external (photoperiod- and vernalization-dependent) and internal (autonomous or age- and gibberellin-dependent) pathways (Greenup et al., 2009; Kim et al., 2009; Amasino, 2010; Zhang et al., 2013). These processes are integrated by at least 60 genes (Kobayashi and Weigel, 2007), including *FLOWERING LOCUS D* (*FD*), *FLOWERING LOCUS E* (*FE*), *FLOWERING WAGENINGEN* (*FWA*), *PROTODERMAL FACTOR2* (*PDF2*), *CONSTANS* (*CO*), *SUPPRESSOR OF OVEREXPRESSION OF CO 1*(*SOC1*), *FLOWERING LOCUS T* (*FT*), *FLOWERING LOCUS C* (*FLC*), *FRIGIDA* (*FRI*), and *VERNALIZATION* (*VRN*) (Samach and Gover, 2001; Castillejo and Pelaz, 2008; Distelfeld et al., 2009; Holefors et al., 2009; Zhang et al., 2013; Fu et al., 2014; Han et al., 2014; Li C. N. et al., 2015; Yruela, 2015). The integrated signal for floral induction is transmitted by the floral meristem identity genes *LEAFY* (*LFY*) and *APETALA1* (*AP1*), after which floral morphogenesis takes place (Komeda, 2004; Qian et al., 2014).

To date, few flowering genes have been cloned from pineapple. A homolog of the *PISTILLATA (PI*) gene, *AcPI*, which plays a crucial role in the regulation of flowering in angiosperms, was isolated from the pineapple cultivar “Comte de Paris”. Its expression level was the highest at 40 days after flower induction, which is when multiple fruits and floral organs form (Lv et al., 2012a). A homolog of the *FLOWERING LOCUS T (FT*) gene, *AcFT*, was also isolated from pineapple. qPCR analysis revealed that its expression was elevated in the flesh and absent in the leaves and that it was expressed at the highest level at 40 days after flower induction (Lv et al., 2012b). In other studies of the mechanism of flowering stimulation by ethephon, three aminocyclopropane carboxylic acid (ACC) synthase genes (*AcACSs*), which play vital roles in the biosynthesis of ethylene, were isolated from pineapple (Botella et al., 2000; Trusov and Botella, 2006; Yuri and José, 2006; Choudhury et al., 2008). Transgenic pineapple plants carrying a silenced *AcACS2* gene showed a marked delay in flowering compared to non-silenced transgenic plants and control non-transformed plants. The *AcACS2* gene may be one of the key contributors to the triggering of natural flowering in mature pineapple under commercial field conditions (Trusov and Botella, 2006; Yuri and José, 2006). Compared with other monocots, such as rice, wheat and cereal, little information is available regarding the role of flowering genes in the regulation of the vegetative to flowering transition and flower initiation in pineapple. In addition, few reports have investigated the genes related to the stimulation of pineapple flowering by ethephon and their functions.

Recently, Illumina sequencing techniques have enabled fascinating discoveries in the life sciences and have dramatically improved the efficiency of gene discovery (Zhang et al., 2013). RNA-seq technology allows for the determination of genome-wide expression levels as well as the identification of novel transcripts and isoforms, and it has been used successfully in evaluations of numerous plant species, including *Arabidopsis* (Zhu et al., 2013), rice (Huang et al., 2014), soybean (Stamm et al., 2014), maize (Dukowic-Schulze et al., 2014; Thakare et al., 2014), and non-model species, such as wild strawberry (Sánchez-Sevilla et al., 2014). With regard to pineapple, Ong et al. (2012) performed transcriptome sequencing of ripe yellow pineapple fruit flesh using Illumina technology to determine the mechanisms and processes underlying fruit ripening. However, there is still a dearth of data on the molecular mechanisms of stimulation of pineapple flowering by ethephon. Further transcriptome sequencing studies of pineapple flowering and related genes induced by ethephon during the floral transition are needed.

Accordingly, in this study, RNA-seq and gene expression profiling were performed using Illumina technology to investigate the flowering pathway. In addition, various screened candidate genes related to flowering transition were analyzed by qPCR. The findings of this study contribute to the understanding of the molecular mechanisms of ethephon-mediated stimulation of the floral transition in pineapple.

## Materials and methods

### Site description

To investigate the molecular mechanisms of the ethephon-mediated induction of the flowering transition in pineapple plants, a potted culture experiment was conducted at an experimental orchard at the Institute of Fruit Tree Research of the Guangdong Academy of Agricultural Sciences located in the Tianhe District (113.35° N, 23.12° E), Guangzhou, Guangdong Province, P. R. China. The local climate is a southern subtropical monsoon climate, with a mean annual precipitation of more than 1800 mm and an average annual air temperature of 21.5~22.2°C. The monthly average temperature is the highest between June and August, at approximately 28~35°C, and it is the lowest between December and February, at approximately 10~15°C.

### Plant materials and treatments

In this study, the pineapple cultivar “Smooth Cayenne,” categorized as Cayenne, was used. In mid-October 2013, thirty pots containing pineapple plants with similar heights and leaf numbers were transported to a plastic shed. Five days later, flowering was induced in the pineapple plants with 100 mL of ethylene solution containing 1.80 mL·L^−1^ (T1) or 2.40 mL·L^−1^ (T2) ethephon (*v/v* 40%), which was applied to the central cups. The same volume of cleaning water was used as a control (Ct). Ten replicates of the two treatments and control were used, resulting in a total of 30 pots. Fifty days later, when the central cups of the flowering-induced plants opened, three shoot apical meristems (a total of 9) were randomly sampled from the T1, T2, and Ct groups, respectively. All of the samples were placed in liquid nitrogen promptly for further investigation.

### RNA extraction

Total RNA was isolated from each sample using a Trizol Kit (Promega, USA), following the manufacturer's instructions. Subsequently, the total RNA was treated with RNase-free DNase I (Takara Bio, Japan) for 30 min at 37°C to remove residual DNA. RNA quality was verified using a 2100 Bioanalyzer (Agilent Technologies, Santa Clara, CA) and was also evaluated by RNase-free agarose gel electrophoresis. The concentration of total RNA was measured using a 2100 Bioanalyzer at 260 and 280 nm. In the process of RNA extraction, two additional extractions were performed for each treatment or control sample. Among the three RNA extractions from each treatment or control, those with a 260/280 nm ratio between 1.8 and 2.0 were considered to have the best quality and were used for subsequent analyses. Equal amounts of RNA from each sample were mixed for the subsequent steps.

### RNA-seq library construction and sequencing

Total RNA was isolated from each of the samples using a Trizol Kit (Promega, USA), following the manufacturer's instructions. Then, the total RNA was treated with RNase-free DNase I (Takara Bio, Japan) to remove residual DNA. Poly (A) mRNA was isolated using oligo-dT beads (Qiagen). Fragmentation buffer was added to the mRNA to generate short fragments (200 nt). First-strand cDNA was synthesized by random hexamer-primed reverse transcription, and then second-strand cDNA was generated using RNase H and DNA polymerase I. The cDNA fragments were purified using a QIAquick PCR Extraction Kit. These purified fragments were then washed with EB buffer for end reparation poly (A) addition and ligated to sequencing adapters. Following agarose gel electrophoresis and extraction of cDNA from the gels, the cDNA fragments (200 ± 25 bp) were purified and enriched by PCR to construct the final cDNA library. The cDNA library was sequenced with an Illumina sequencing platform (Illumina HiSeq™2000) using paired-end technology. The processing of the original image to determine the sequences, in addition to the base calling and quality value calculations, were performed using Illumina GA Pipeline (version 1.6), from which 90 bp paired-end reads were obtained (Li et al., 2013).

### Illumina read processing and assembly

A Perl program was written to select clean reads by removing low-quality sequences (over 50% of bases with a quality score of lower than 20 in one sequence), reads with more than 5% N bases (unknown bases) and reads containing adaptor sequences. Then, the clean reads were assembled using Trinity to construct unique consensus sequences.

### Functional annotation and classification

The filtered transcripts were annotated using BLASTx (Altschul et al., 1997), which is a tool used to evaluate the similarity between two sequences, against the databases Nr (http://www.ncbi.nlm.nih.gov/), COG (http://www.ncbi.nlm.nih.gov/COG/; Tatusov et al., 2003), KEGG (Kanehisa et al., 2008), and SwissProt (http://www.expasy.ch/sprot; Magrane and Consortium, 2011), with an *e*-value cut-off of < 10^−5^. The protein sequences in the databases with the highest similarity scores were used to obtain functional annotations of the related Unigenes. Blast2GO (Conesa et al., 2005) was used for functional annotation of the Unigenes using the Gene Ontology (GO) database (http://www.geneontology.org/; Ashburner et al., 2000), which contains a series of terms grouped into three ontologies (molecular function, biological process and cellular component) that describe genes. Next, WEGO (http://wego.genomics.org.cn/cgi-bin/wego/index.pl; Ye et al., 2006), which is a statistical tool, was used to classify the GO annotation results. The HMMER3.0 program was used to annotate the unigenes using the Pfam (protein families) database (http://pfam.sanger.ac.uk/).

### Determination of gene expression levels

The sequencing reads were mapped to reference sequences using SOAPaligner/soap2 (Ernst and Bar-Joseph, 2006). Reads that could be uniquely mapped to a gene were used to calculate expression levels. Gene expression levels were measured according to the number of uniquely mapped reads per kilobase of exon region per million mapped reads (RPKM) using the following equation:

$$\begin{array}{l} \text{RPKM}=\frac{10^{6}C}{NL∕10^{3}} \end{array}$$

in which *C* is the number of reads uniquely mapped to a given gene, *N* is the number of reads uniquely mapped to all genes, and *L* is the total length of the exons in the given gene. For genes with more than one alternative transcript, the longest transcript was selected to calculate the RPKM. The RPKM method eliminates the influences of different gene lengths and sequencing discrepancies on gene expression calculations. Therefore, RPKM values can be used directly for comparing differences in gene expression among samples.

### Variations in gene expression

To identify the differentially expressed genes (DEGs) among T1, T2, and Ct, the number of clean tags for each gene was calculated, and the genes that were differentially expressed were identified based on the method described by Audic and Claverie (1997) for determining the threshold *p*-value, FDR and fold change (log${}_{2}^{\text{Ratio}}$). DEGs were defined as those with an FDR of ≤ 0.001 and an absolute value of log${}_{2}^{\text{Ratio}}\geq$ 1.

### Go and pathway enrichment analyses of DEGs

To determine the main biological functions of the DEGs, they were first annotated using the Gene Ontology (GO) database (http://www.geneontology.org/) and Blast2GO (Conesa et al., 2005) according to their numerical order in the nr database. Blast2GO is an all-in-one tool for the functional annotation of novel sequences and analysis of annotation data. It has been cited by other articles over 150 times and is a widely recognized GO annotation software program. After obtaining GO annotations for each of the DEGs, WEGO software (Ye et al., 2006) was used to obtain GO functional classifications. GO enrichment analysis was performed using hypergeometric testing to identify significantly enriched GO terms among the DEGs compared to the genome background.

### Validation of subset of DEGs by qPCR

Total RNA was extracted as described above. Each RNA sample was treated with RNase-free DNase (Takara) following the manufacturer's protocol to remove any residual genomic DNA (gDNA). DNase-treated RNA (2 mg) was subjected to reverse transcription using arbitrary primers and PrimeScript™Reverse Transcriptase (Takara) according to the manufacturer's protocol. The gene expression levels were determined using Ct values with the formula 2^−ΔCt^. The actin gene of *A. comosus* (HQ148720.1) was used as a reference gene. Each qPCR analysis was performed in triplicate. The sequences of the specific primer sets are listed in Additional File 1.

## Results

### DGE library sequencing and evaluation

Shoot apices were sampled from the Ct, T1, and T2 plants and were sequenced to construct three digital gene expression (DGE) libraries. The results of the sequencing quality assessment and alignment analysis are presented in Table 1. Low-quality reads, including adaptor reads and reads containing Ns, represented 1.27, 1.24, and 1.24% of the reads in the three libraries, respectively.

**Table 1:** **DGE sequencing quality assessment and statistics of alignment analysis**.

**Terms**	**Ct**	**T1**	**T2**
Total Reads	20,732,818	23,720,082	21,372,324
Total Reads (%)	100.00%	100.00%	100.00%
Only adaptor reads	132,332	148,286	133,181
Only adaptor reads (%)	1.26%	1.23%	1.23%
Reads containing Ns	0	0	0
Reads containing Ns (%)	0.00%	0.00%	0.00%
Low-quality reads	548	642	623
Low-quality reads (%)	0.01%	0.01%	0.01%
Total clean reads	10,366,409	11,860,041	10,686,162
Total clean reads (%)	98.73%	98.76%	98.76%
Total Base Pairs	2,073,281,800	2,372,008,200	2,137,232,400
Total Base Pairs (%)	100.00%	100.00%	100.00%
Total Mapped Reads	16,889,894	19,132,922	17,367,263
Total Mapped Reads (%)	81.46%	80.66%	81.26%
Perfect match	13,559,314	15,274,836	13,877,373
Perfect match (%)	65.40%	64.40%	64.93%
≤ 2 bp mismatch	3,330,580	3,858,086	3,489,890
≤ 2 bp mismatch (%)	16.06%	16.27%	16.33%
Unique match	10,223,609	11,745,964	10,541,902
Unique match (%)	49.31%	49.52%	49.33%
Multi-position match	6,666,285	7,386,958	6,825,361
Multi-position match (%)	32.15%	31.14%	31.94%
Total Unmapped Reads	3,842,924	4,587,160	4,005,061
Total Unmapped Reads (%)	18.54%	19.34%	18.74%

### Sequence assembly

To broadly investigate the genes associated with floral induction in pineapple, an overview of the pineapple transcriptome was obtained, which is summarized in Table 2. A cDNA library was prepared using a mixture of equal amounts of RNA isolated from the stem apices of Ct, T1, and T2 plants, and paired-end sequences were generated using an Illumina Hiseq™2000 platform. After the cleaning and removal of low-quality reads (quality scores < 20), 65,825,224 clean reads with an average length of 100 bp were identified in the three libraries (66,656,448 raw reads), with a Q20 percentage (base quality score of over 20) of 98.03%. The GC percentage was 50.15%.

**Table 2:** **Summary of pineapple transcriptome**.

Total number of raw reads	66,656,448
Total number of clean reads	65,825,224
Total clean nucleotides (nt)	6,582,522,400
Q20 percentage	98.03%
N percentage	0.00%
GC percentage	50.15%
Average read length	100 bp
Total number of contigs	148, 728
Mean length of contigs	1225 bp
N50 size	2023 bp
Total number of unigenes	129, 594
Mean length of unigenes	1173 bp

**Figure 1:**
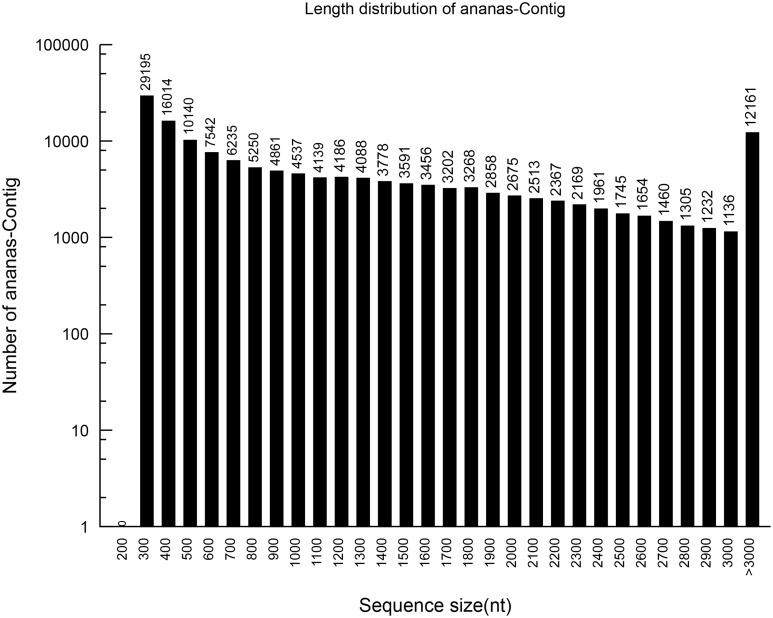
**The length distribution of the assembled pineapple contigs**.

**Figure 2:**
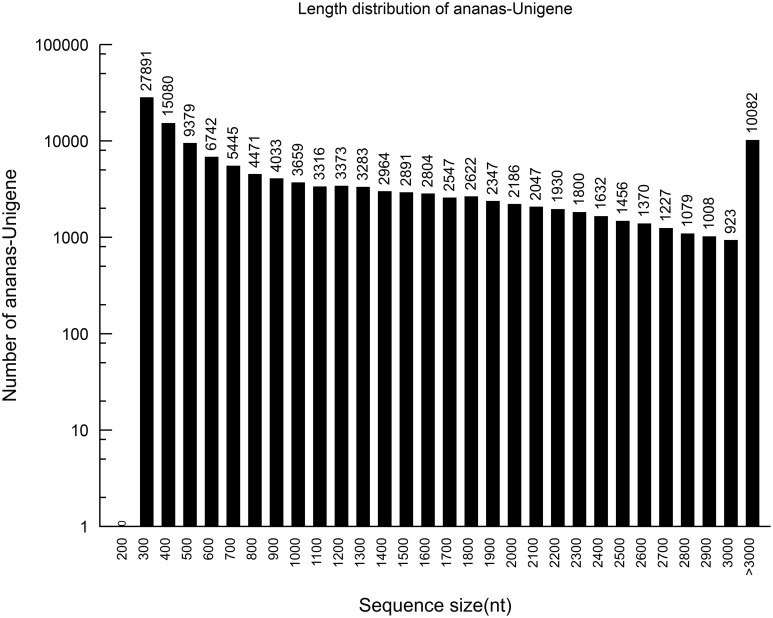
**The length distribution of the assembled pineapple unigenes**.

Based on the high-quality reads, 148,728 contigs were assembled, with 6,582,522,400 bp and an N50 of 2023 bp. The sizes and length distributions of the contigs and unigenes are presented in Figures 1, 2. The lengths of the contigs ranged from 201 to 11,724 bp, with an average length of 1225 bp. A total of 1225 contigs were >500 bp, and 2038 were between 400 and 500 bp in length. Most of the contigs were between 75 and 400 bp. Among them, 93,379 were between 300 and 400 bp in length, which represented 62.76% of the total contigs. However, 10,140 contigs (6.82%) were between 200 and 300 bp, 16, 014 (10.77%) were between 100 and 200 bp, and 29,195 (19.63%) were between 75 and 100 bp.

Using the paired-end reads, these contigs were further assembled into 129,594 unigenes by Trinity. The lengths of the unigenes ranged from 201 to 12,421 bp, with an average of 1173 bp.

### Functional annotation

The statistical results for annotation of the pineapple unigenes are summarized in Table 3. A search of the reference sequences and a BLASTX search against nr, SwissProt, COG, KEGG, GO and Pfam databases produced significant results for 129,594 unigenes.

**Table 3:** **Summary of unigene annotations (*e* < 10^−5^)**.

**Database**	**Number of unigenes**	**Percentage (%)**
nr	75,592	58.33
SwissProt	60,909	47.00
COG	36,523	28.18
KEGG	24,775	19.12
GO	35,485	27.38
Pfam	57,985	44.74
Total unigenes	129,594	100.00

Among the 129,594 unigenes, 75,592 (approximately 58.33%) could be annotated in nr based on sequence homology, 60, 909 (47.00%) could be annotated in SwissProt, 24,775 (19.12%) could be annotated in KEGG, 36,523 (28.18%) could be annotated in COG, and 35,485 (27.38%) could be annotated in GO. In addition, 57,985 (44.74%) of the unigenes could be annotated in the Pfam database.

**Figure 3:**
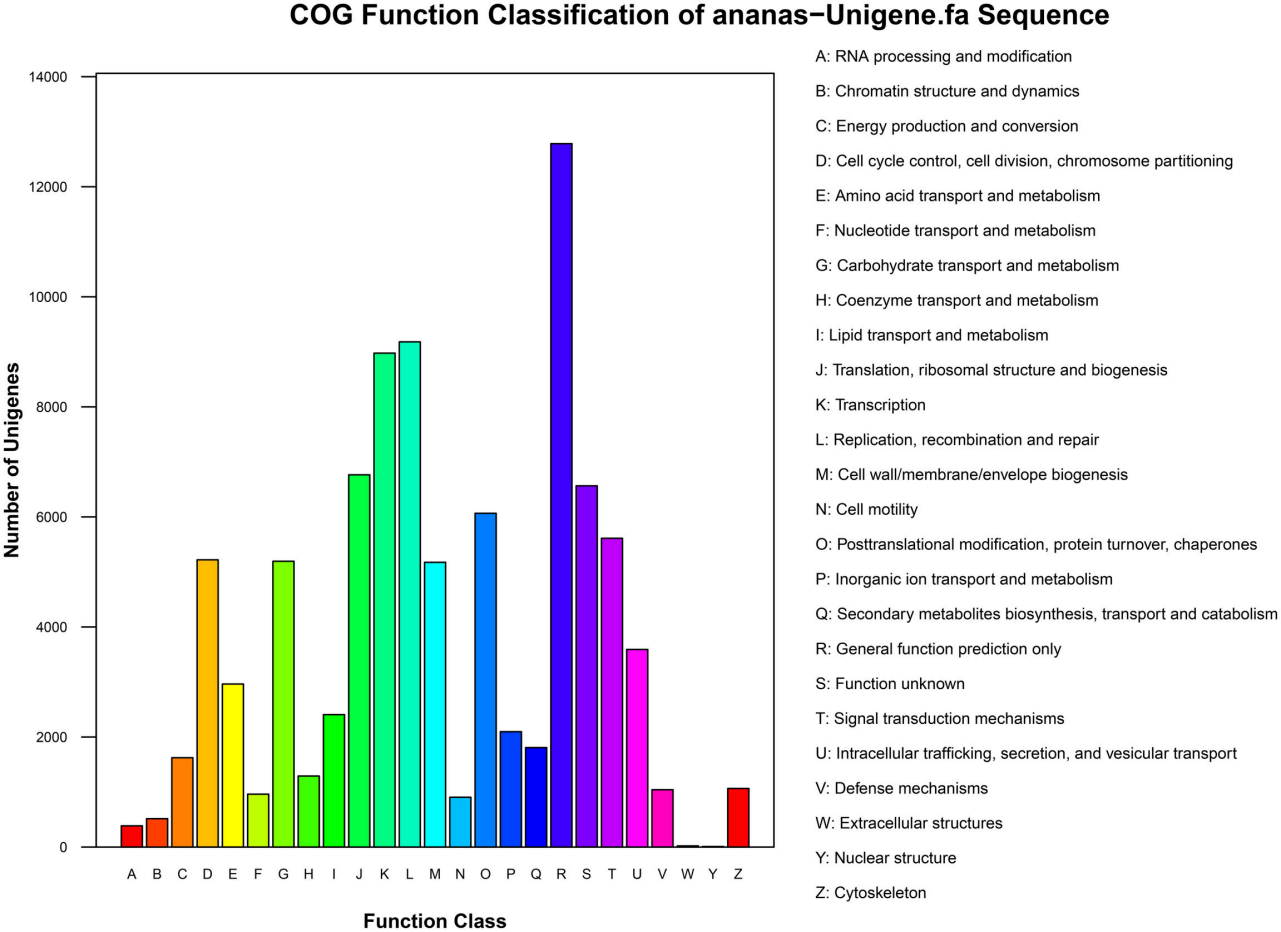
**COG functional annotation of pineapple unigenes**.

The results of COG functional annotation of the pineapple unigenes are shown in Figure 3. A total of 35,523 unigenes were classified into 25 COG functional categories, among which the category “General function prediction” was predominant. A high percentage of genes were also assigned to “Transcription,” “Replication, recombination and repair,” and “Translation, ribosomal structure and biogenesis.” Only a few genes were assigned to the categories “Extracellular structures” and “Nuclear structure” (Additional File 2).

COG analysis revealed that the identified genes are involved in various biological processes. For example, 5612 unigenes were annotated to “Signal transduction mechanisms” and are thus involved in signal transduction pathways, particularly the transduction of plant hormones; in addition, 5194 were annotated to “Carbohydrate transport and metabolism” and are therefore associated with carbohydrate transport and metabolism pathways, and 5220 were annotated to “Cell cycle control, cell division, and chromosome partitioning” and are involved in cell division and chromosome partitioning. Pathways associated with the transduction of plant hormones, carbohydrate transport and metabolism, cell division, and chromosome partitioning are involved in floral induction. Thus, identification of the genes in these pathways is crucial and will aid in the elucidation of the molecular mechanisms of floral induction by ethephon in pineapple.

### GO classifications of unigenes

**Figure 4:**
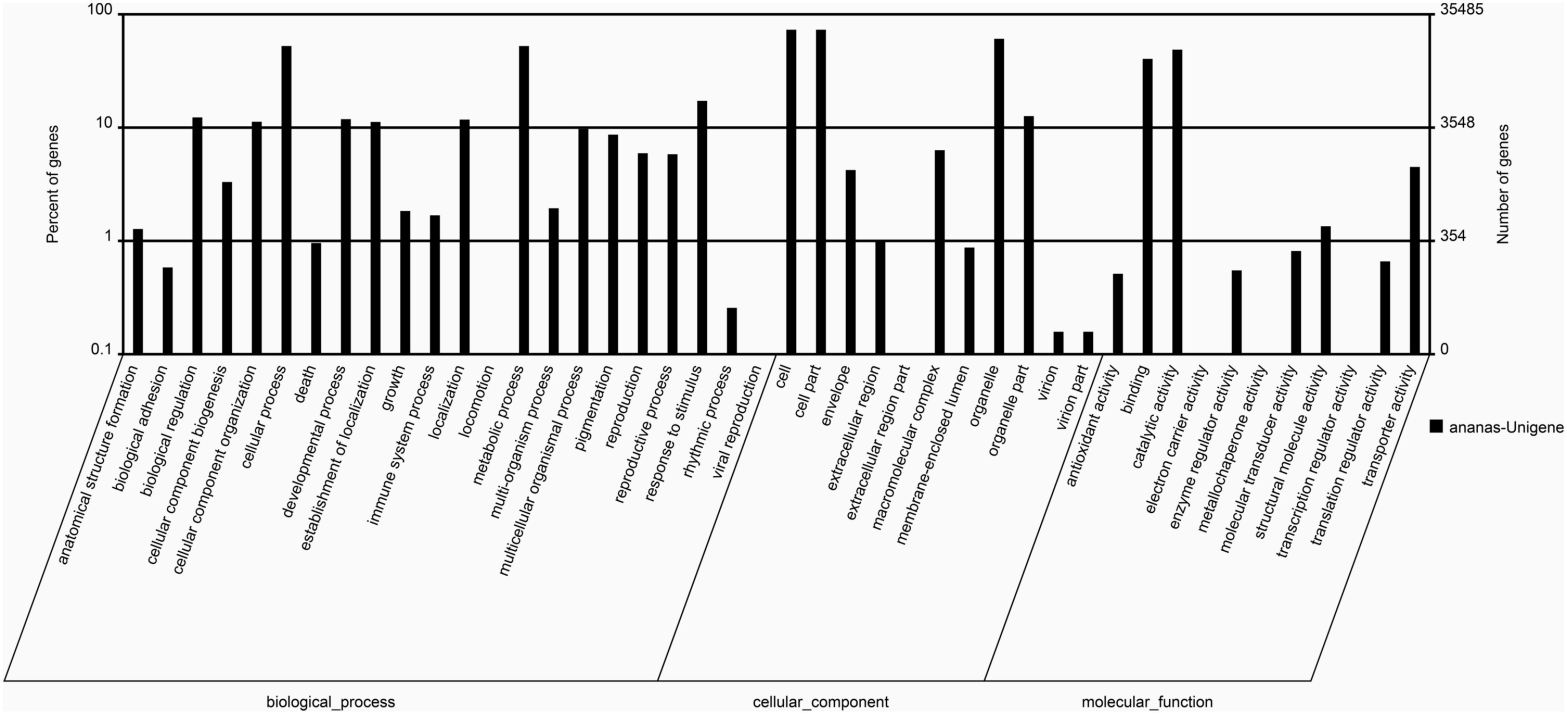
**The main enriched GO classifications of the assembled unigenes**.

In this study, GO analysis was performed to categorize the functions of the predicted genes in pineapple, and the predominant GO categories among the assembled unigenes are presented in Figure 4. A total of 35,485 annotated genes were analyzed and divided into 44 functional GO categories within the three main ontologies of biological processes, cellular component, and molecular function. Cellular and metabolic processes were the most highly represented GO categories in the biological process ontology, with 18,640 and 18,627 unigenes, respectively. In addition, 6115 unigenes were annotated to the category “Response to stimulus,” and 2109 and 2066 unigenes were annotated to “Reproduction” and “Response to reproduction,” respectively. The GO annotations of these unigenes may aid in the identification of the molecular mechanisms of floral induction by ethephon in pineapple.

The GO categories “Cell,” “Cell part,” and “Organelle” were predominant in the cellular component ontology, with 25,950, 25,950, and 21,578 unigenes, respectively. These unigenes may be associated with cell cycle control, cell division, and chromosome partitioning. The identification of these unigenes may be useful for understanding the molecular mechanisms of floral induction in pineapple by ethephon.

In the “molecular function” ontology, a high percentage of unigenes were annotated to the categories “Catalytic activity” and “Binding” (49.9 and 41.4%, respectively). Only a few unigenes were annotated to “Electron carrier activity” (16), “Metallochaperone activity” (1), and “Transcription regulator activity” (20).

### KEGG pathway assignments

**Figure 5:**
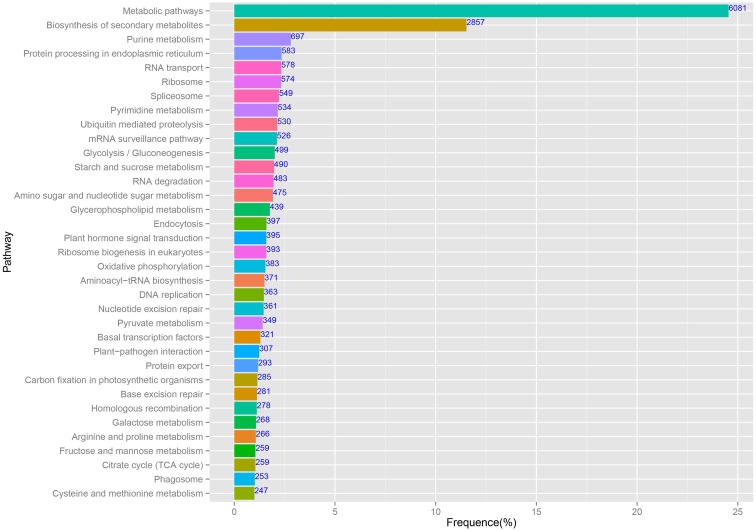
**The main enriched KEGG pathways of the assembled unigenes**.

The predominant KEGG pathways of the assembled unigenes are presented in Figure 5. In this study, 65,535 annotated sequences were mapped to reference pathways described in KEGG. In total, 24,775 unique sequences were assigned to 122 KEGG pathways (Additional File 3). Among the unigenes, the highest percentage (6081/24,775, 24.54%) was assigned to “Metabolic pathways,” followed by “Biosynthesis of secondary metabolites” (2857/24,775, 11.53%). These pathway assignments provide a valuable resource for investigating specific processes, functions, and pathways in pineapple.

### Identifying pineapple flowering-associated genes

Based on the annotations, a few pineapple genes associated with flowering time were identified, including photoperiod pathway genes, such as *EARLY FLOWERING 3* (*ELF3*), *CIRCADIAN TIMEKEEPER* (*CTK*), and *COSTANS* (*CO*); vernalization pathway genes, such as *VERNALIZATION* (*VRN1*), *FRIGIDA* (*FRI*), and MADS-box genes; floral integrator pathway genes, such as *CAULIFLOWER* (*CAL*), *AP2, FT*, and *TERMINAL FLOWER 1* (*TFL1*); and the floral meristem identity genes *LFY, AGMOUS* (*AG*), and *AP1*. These results provide important insights into the mechanism of ethephon-mediated floral induction, floral development, and flower organ formation in pineapple.

### Variations in gene expression

**Figure 6:**
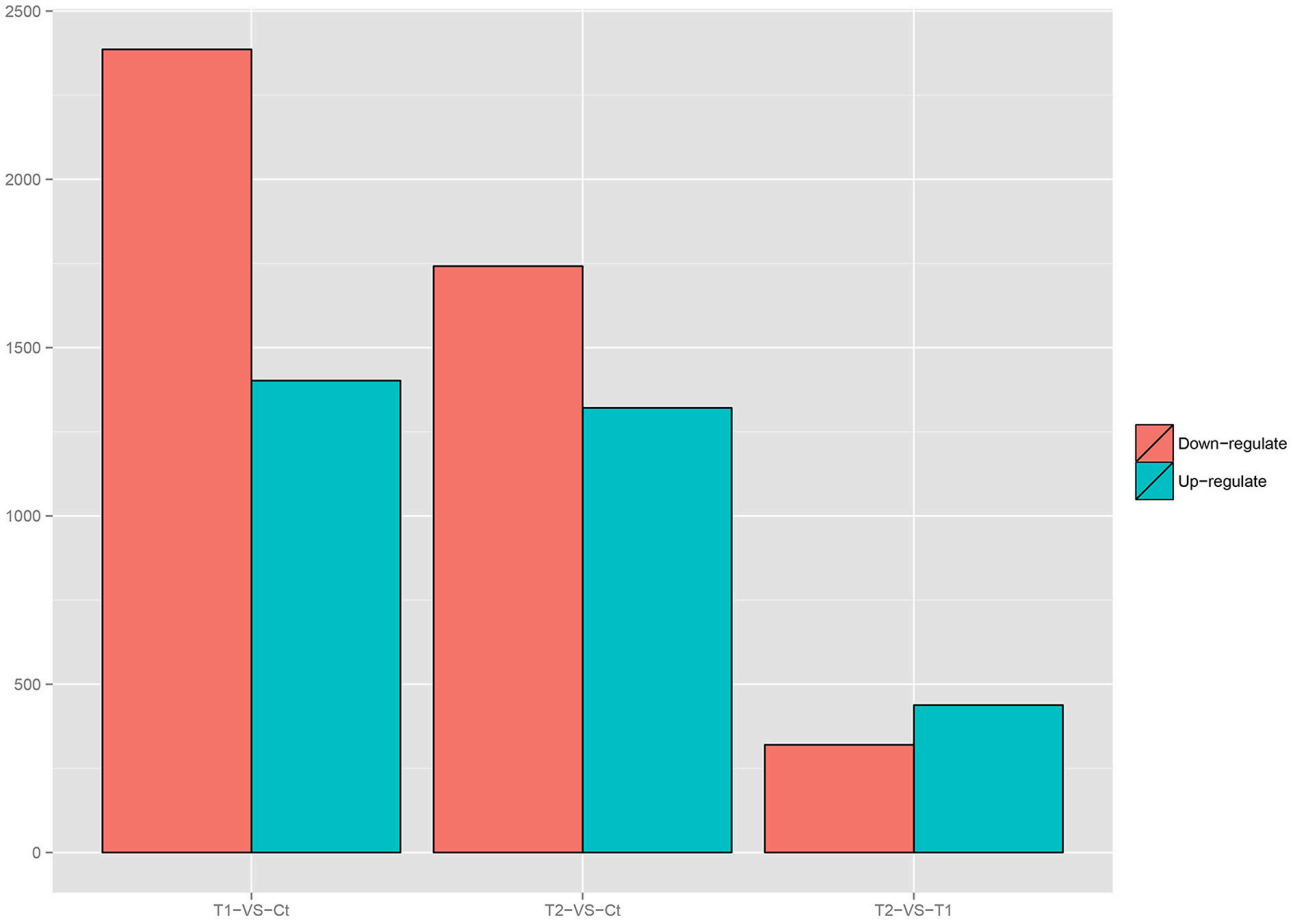
**Number of DEGs in each comparison**.

Variations in gene expression were examined by comparing the T1 and Ct, T2 and Ct, and T1 and T2 groups. The numbers of DEGs in each comparison are presented in Figure 6. The comparison of T1 and Ct, as well as that of T2 and Ct, revealed significant differences in gene expression. A total of 3788 DEGs were identified in T1 compared to Ct, including 1402 up-regulated and 2386 down-regulated genes. In addition, a total of 164 DEGs were specifically expressed in T1, and 211 were specifically expressed in Ct. Further, 3062 DEGs were identified in T2 compared to Ct, including 1321 up-regulated and 1741 down-regulated DEGs. A total of 77 DEGs were specifically expressed in T2, and 116 were specifically expressed in Ct. Finally, 758 DEGs were detected in T2 compared to T1. Among them, 438 were up-regulated, and 320 were down-regulated. A total of 24 DEGs were specifically expressed in T2, and 105 were specifically expressed in T1. The DEGs that were specifically expressed in either the T1, T2 or Ct group are listed in Additional Files 4–6.

### GO classifications of DEGs

**Figure 7:**
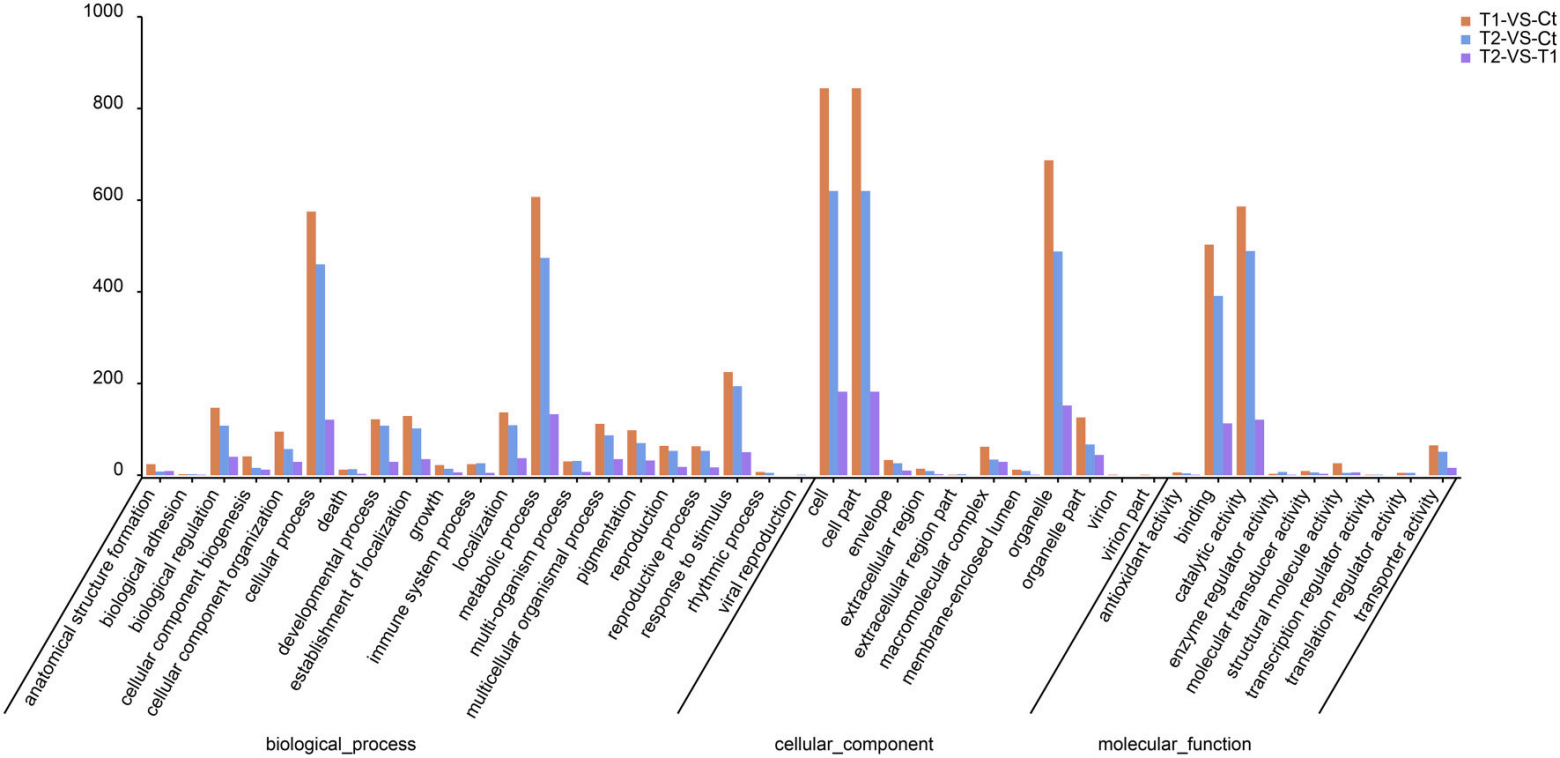
**GO classifications of top DEGs**.

**Figure 8:**
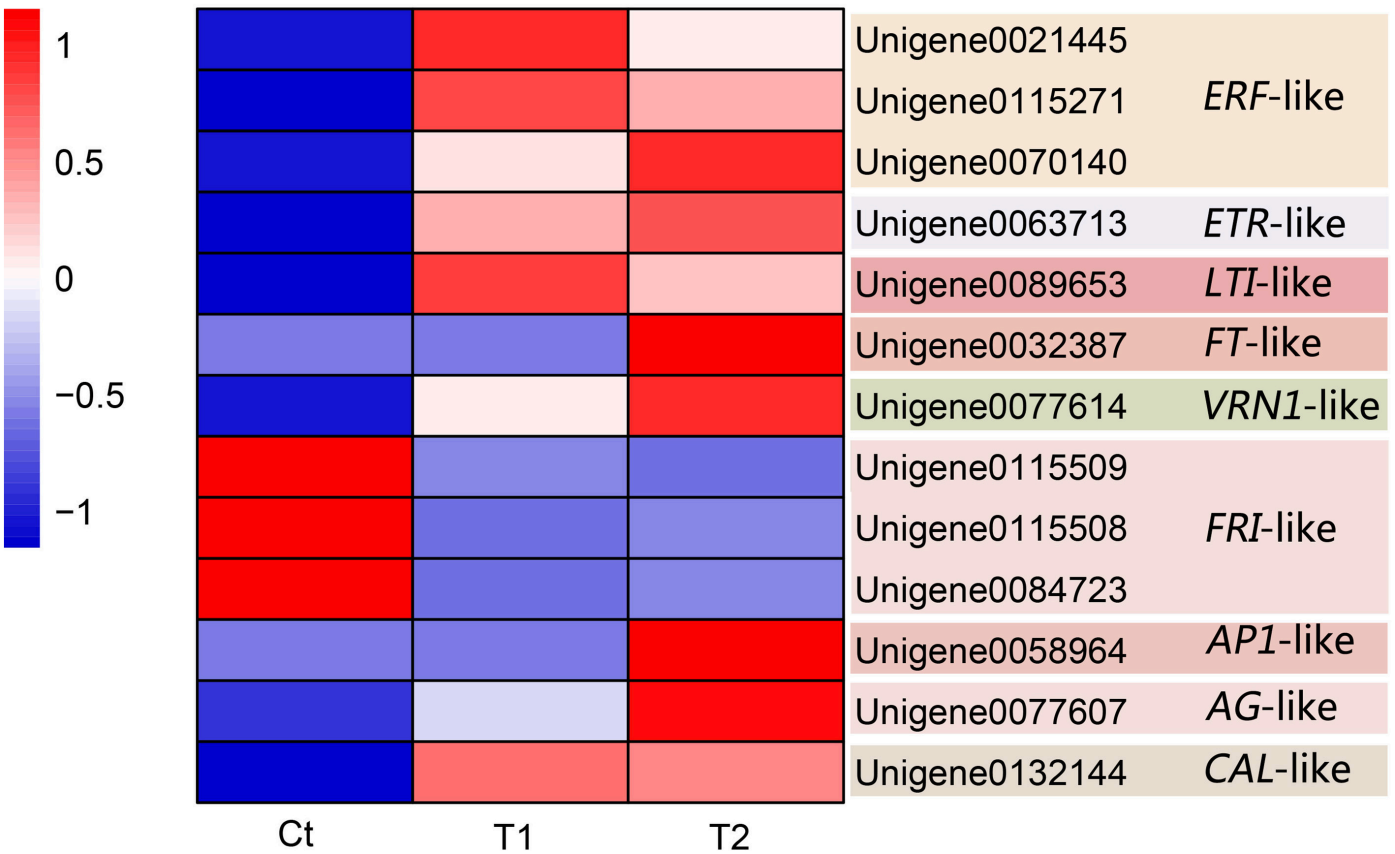
**Candidate DEGs related to pineapple floral induction by ethephon**.

The GO classifications of the top DEGs are presented in Figure 7. In the biological process ontology, most of the DEGs were annotated to the metabolic process and cellular process GO categories (Figure 8). In total, 607 and 575 DEGs were annotated to metabolic process and cellular process, respectively, in T1 compared to Ct, in addition to 474 and 460, respectively, in T2 compared to Ct, and 133 and 121, respectively, in T2 compared to T1.

In the cellular component ontology, most of the DEGs were annotated to the categories cell, cell part and organelle (Figure 7). In total, 844, 844, and 687 DEGs were annotated to cell, cell part and organelle, respectively, in T1 compared to Ct, in addition to 620, 620, and 488, respectively, in T2 compared to Ct, and 182, 182, and 152, respectively, in T2 compared to T1.

In the molecular function ontology, most of the DEGs were annotated to the catalytic activity and binding categories (Figure 7). In total, 586 and 503 DEGs were annotated to catalytic activity and binding in T1 compared to Ct, in addition to 489 and 391, respectively, in T2 compared to Ct, and 121 and 113, respectively, in T2 compared to T1.

### KEGG classifications of DEGs

KEGG pathway analysis was performed to further examine the DEGs. In the comparison of T1 with Ct, 17 related pathways were found to be significantly enriched (Additional File 7). Several interesting and important pathways were enriched among the DEGs, and the top pathways included “Metabolic pathways” (ko01100), “Biosynthesis of secondary metabolites” (ko01110), “Plant hormone signal transduction” (ko04075), “Oxidative phosphorylation” (ko00190) and “Plant-pathogen interaction” (ko04626). In the comparison of T2 with Ct, 20 related pathways were found to be significantly enriched (Additional File 8), including “Metabolic pathways” (ko01100), “Biosynthesis of secondary metabolites” (ko01110), “Protein processing in endoplasmic reticulum” (ko04141), “Plant hormone signal transduction” (ko04075), and “Phenylpropanoid biosynthesis” (ko00940). In the comparison of T2 with T1, significantly enriched pathways (Additional File 9) included “Metabolic pathways” (ko01100), “Oxidative phosphorylation” (ko00190), and “Phagosome” (ko04145).

### Candidate DEGs related to pineapple floral induction by ethephon and qPCR validation

Thirteen DEGs were identified as candidate DEGs involved in the process of floral induction by ethephon in pineapple. Figure 8 and Table 4 summarize the expression of these thirteen DEGs.

**Table 4:** **List of the candidate DEGs involved in floral induction by ethephon according to RNA-seq data**.

**Gene identifier**	**Gene description**	**Fold change (log**${}_{2}^{\text{Ratio}}$**)**		**FDR**	
		**T1-vs-Ct**	**T2-vs-Ct**	**T1-vs-Ct**	**T2-vs-Ct**
Unigene0021445	Ethylene-responsive factor (*ERF*)	1.36^*^	0.90	0^**^	0^**^
Unigene0115271	Ethylene-responsive factor (*ERF*)	1.13^*^	0.92	2.19E-11^**^	1.21E-11^**^
Unigene0070140	Ethylene-responsive factor (*ERF*)	1.93^*^	2.54^*^	0.002	9.44E-05^**^
Unigene0063713	Ethylene receptor (ETR)	1.51^*^	1.75^*^	0.002	0.00014^**^
Unigene0089653	Low-temperature-induced protein (*LTI*)	12.74^*^	12.25^*^	3.82E-06^**^	0.00035^**^
Unigene0032387	Flowering locus T-like protein (*FT*-like)	NF	12.08^*^	NF	2.34E-06^**^
Unigene0077614	Vernalization protein (*VRN1*)	15.69^*^	16.55^*^	1.42E-14^**^	0^**^
Unigene0115509	Protein FRIGIDA-like (*FRI*)	−2.46^*^	−2.91^*^	4.87E-107^**^	1.05E-120^**^
Unigene0115508	Protein FRIGIDA-like (*FRI*)	−2.15^*^	−1.83^*^	8.03E-8^**^	6.40E-6^**^
Unigene0084723	Protein FRIGIDA-like (*FRI*)	−1.24^*^	−1.17^*^	4.51E-19^**^	2.05E-16^**^
Unigene0058964	AP1-like MADS box transcription factor (*AP1*)	NF	14.73^*^	NF	0^**^
Unigene0077607	Agamous-like MADS-box protein (*AG*)	3.52^*^	4.89^*^	9.75E-05^**^	0.00016^**^
Unigene0132144	Truncated transcription factor CAULIFLOWER A-like (*CAL*)	11.41^*^	11.30^*^	1.34E-13^**^	3.39E-12^**^

* *absolute value of log${}_{2}^{Ratio}$ ≥ 1*,

** FDR ≤ 0.001, NF, not found.

### *ERF*-like and *ETR*-like

Three differentially expressed *ERF*-like (ethylene–responsive factor) genes, namely Unigene0021445, Unigene0115271, and Unigene0070140, were isolated and identified as candidate genes (Figure 8 and Table 4). Among them, Unigene0021445 and Unigene0115271 were identified in the comparison of T1 with Ct and were found to be up-regulated by 1.36- and 1.13-fold, respectively. Unigene0070140 was identified in the comparison of T2 with Ct and was found to be up-regulated by 2.54-fold.

A differentially expressed *ETR*-like (ethylene receptor) gene, Unigene0063713, was identified as a candidate gene in the T2 group and was found to be up-regulated by 1.75-fold (Figure 8 and Table 4). This gene was also up-regulated by 1.51-fold in the T1 group but was not considered to be differentially expressed because of the high FDR value (0.001977). These results indicate that floral stimulation by ethephon induces expression of the *ETR* gene.

### *LTI*-like

In this study, a differentially expressed *LTI*-like (low temperature-induced) gene, Unigene0089653, was isolated and identified as a candidate gene. The expression of this gene was induced by the T1 and T2 treatments, which resulted in 12.74- and 12.25-fold up-regulation, respectively (Figure 8 and Table 4). These results showed that the treatment of pineapple with ethephon induced the expression of an *LTI*-like gene.

### *FT*-like

Three *FT*-like genes were isolated and identified (Unigene0109587, Unigene0032386, and Unigene0032387), and their expression was found to be up-regulated in the T1 and T2 groups compared with the Ct group. In particular, the *FT*-like gene Unigene0032387 was identified as a DEG in the comparison of T2 with Ct, exhibiting 12.08-fold increased expression, and it was screened as a candidate gene (Figure 8 and Table 4).

### *VRN1*-like

A *VRN1*-like gene, Unigene0077614, was isolated and identified as a DEG. Ethephon treatment induced the expression of this gene, which exhibited 15.69- and 16.55-fold up-regulation in the T1 and T2 groups, respectively (Figure 8 and Table 4); thus, it was screened as a candidate gene.

### *FRI*-like

Three differentially expressed *FRI*-like genes, namely Unigene0115509, Unigene0115508, and Unigene0084723, were isolated and identified as candidate genes (Figure 8 and Table 4). The expression of these genes was down-regulated by 2.46-, 2.15-, and 1.24-fold, respectively, in the T1 group (Figure 8 and Table 3). Similarly, their expression was down-regulated by 2.91-, 1.83-, and 1.17-fold, respectively, in the T2 group (Figure 8 and Table 4).

### Floral meristem identity (FMI) genes

#### *AP1*-like

An *AP1*-like gene, Unigene0058964, was isolated and identified as a DEG in the comparison of T2 with Ct. Its expression was up-regulated by 14.73-fold after ethephon stimulation in the T2 group (Figure 8 and Table 4).

#### *CAL*-like

A *CAL*-like gene, Unigene0132144, was isolated and identified as a DEG. Its expression was up-regulated in the T1 and T2 groups by 11.41- and 11.30-fold, respectively (Figure 8 and Table 4).

#### *AG*-like

One *AG*-like gene, Unigene0077607, was isolated and identified as a DEG. Its expression was up-regulated in the T1 and T2 groups by 3.52- and 4.89-fold, respectively (Figure 8 and Table 4).

**Figure 9:**
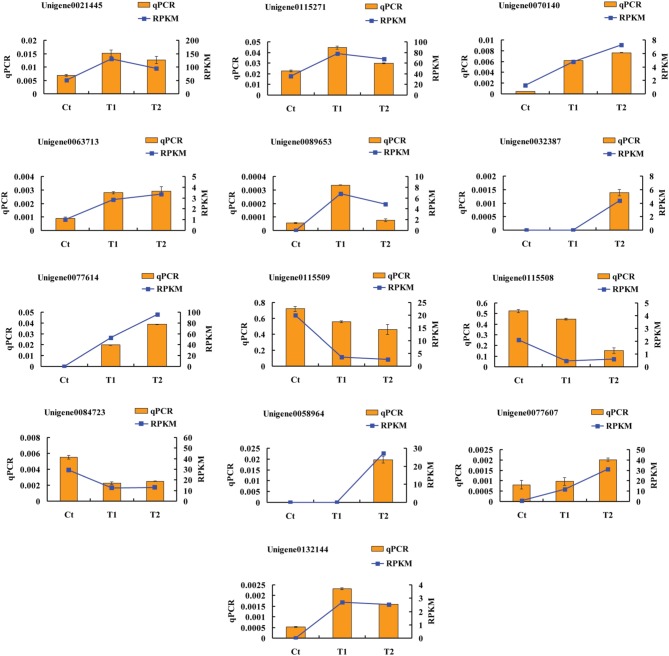
**Expression levels of candidate DEGs determined using by qPCR and RNA-seq**.

To validate the accuracy and reproducibility of the transcriptome analysis results, qPCR was performed to assess the thirteen DEGs described above. Figure 9 shows the expression levels of the candidate DEGs determined using qPCR and RNA-seq. The expression profiles of the thirteen genes revealed by qPCR were consistent with the corresponding RPKM values derived from RNA-seq.

## Discussion

### Illumina sequencing of pineapple and sequence annotation

The pineapple is an economically important tropical fruit with a delicate taste, and it has widespread consumer acceptance in both fresh and processed forms (Pino and Queris, 2010). Despite its economic importance, little research has been conducted on the genetics of this crop fruit because of the low number of pineapple genome sequences that are available in public databases. In a previous study, Ong et al. (2012) generated 4.7 million Illumina paired-end reads that were then assembled into 28,728 unique transcripts. This group performed NCBI searches and GO and KEGG pathway analyses and revealed the complete transcriptomic profile of pineapple.

In this study, RNA-seq and differential gene expression profiling analyses were performed using Illumina sequencing, which generated 65,825,224 clean reads that were assembled into 129,594 unigenes with an average sequence length of 1173 bp. The numbers of high-quality reads and assembled unigenes surpassed those of the previous study (Ong et al., 2012). The results of this study are strong, as the pineapple unigenes were assembled using only shoot apical meristem tissue, and the unigenes were assembled without the use of a reference genome. The unigenes assembled in this study were subjected to a BLASTx similarity search and annotation against the nr, SwissProt, COG, KEGG, GO, and Pfam databases. A total of 19.12–58.33% of the unigenes were annotated, suggesting that the flower transition of pineapple involves many unique processes and pathways. Similar results have been found in studies of the transcriptome of an Orchidaceae species associated with floral development and the response of cassava to cold stress (An et al., 2012; Zhang et al., 2013). The COG functional annotation of the unigenes revealed that the category “General function prediction” was predominant among the 25 functional categories identified. A large number of unigenes were also annotated to the category “Signal transduction mechanisms.” Similar results have been obtained in studies of genes associated with floral development in Orchidaceae and Bambusoideae (Zhang et al., 2012, 2013). In addition, a large number of unigenes were annotated to the categories “Carbohydrate transport and metabolism” and “Cell cycle control, cell division, and chromosome partitioning.” Pathways involving carbohydrate transport and metabolism and cell division and chromosome partitioning are involved in plant floral induction (Rady and El-Yazal, 2013; Vaddepalli et al., 2015). These results provide a foundation for further studies of gene expression and the genomics of pineapple in relation to flower induction and reproductive growth.

### Differential gene expression profiling related to flower induction

In this study, DEGs between each of the treatments and control were identified. More DEGs were identified in the comparisons of T1 and T2 with Ct, and only a few were detected in the comparison of T1 with T2. Most of the enriched KEGG pathways identified in the comparison of T1 with Ct were similar to those detected in the comparison of T2 with Ct. This result suggests that the two ethephon treatments (T1 and T2) have common functions in the induction of pineapple flowering. GO analysis of the transcriptomes revealed that the DEGs were predominantly annotated to subcategories such as metabolic process, cellular process, cell, cell part, organelle, and catalytic activity and binding. Similar results have been obtained in studies of differential gene expression in an Orchidaceae species in response to floral development, as well as in litchi in response to reactive oxygen species (Zhang et al., 2013; Lu et al., 2014). KEGG pathway analysis revealed that the pathways “Metabolic pathways” (ko01100), “Biosynthesis of secondary metabolites” (ko01110), and “Plant hormone signal transduction” (ko04075) were significantly enriched among the DEGs. Similar results have been obtained in studies of differential gene expression associated with floral development in Bambusoideae species, *Paphiopedilum* orchid, longan, and *Tapiscia sinensis* (Zhang et al., 2012; Lai and Lin, 2013; Li D. M. et al., 2015; Zhou et al., 2015). The DEGs assigned to the pathway “Plant hormone signal transduction” are involved in the signal transduction of several plant growth regulators, auxin, cytokinin, gibberellin, abscisic acid, ethylene, brassinosteroid, jasmonic acid (JA), and salicylic acid (SA) and thus regulate plant floral induction, reproductive growth and stimulus responses (Aloni et al., 2006; Li L. F. et al., 2015; Vaddepalli et al., 2015). These results indicate that ethephon-mediated stimulation of floral induction in pineapple activates “Plant hormone signal transduction” pathways. This information provides a foundation for further identification of pineapple floral induction-associated genes.

### Response of pineapple floral induction-associated genes to stimulation by ethephon

In general, pineapple flowering induction is triggered by a small burst of ethylene production (ethephon solution) applied to the central cup (Trusov and Botella, 2006; Van de Poel et al., 2009). ACS (ACC synthase), which is the rate limiting enzyme in the ethylene biosynthesis pathway, is induced by exogenous application of ethylene in pineapple, and the *AcACS2* gene is thought to be one of the key contributors to the triggering of natural flowering in mature pineapple (Botella et al., 2000; Trusov and Botella, 2006; Yuri and José, 2006; Choudhury et al., 2008). In this study, an *ACS*-like gene of pineapple was isolated, and RPKM analysis indicated that this gene was induced by ethephon stimulation. However, it was not considered to be differentially expressed because of the high *P*-value and FDR value. Further, three *ERF*-like (ethylene–responsive factor) genes and one *ETR*-like (ethylene receptor) gene were found to be differentially expressed, exhibiting up-regulation in response to ethephon stimulation. These results are in agreement with those of previous studies of the expression of *ETR* genes in cut rose during flower opening (Ma et al., 2006) and of the expression of *ERF* genes in rice during flowering (Hu et al., 2008) in response to exogenous ethylene. It is known that the expression of certain *ERF* genes is induced by ethylene, jasmonic acid (JA), abscisic acid (ABA), and environmental stress, suggesting that *ERF* proteins could play important roles in the processes regulated by these signaling pathways (Fujimoto et al., 2000; Gutterson and Reuber, 2004; Jin and Liu, 2008; Shinshi, 2008). Auxin has also been reported to promote ethylene synthesis, and both are related to the switch of pineapple from the vegetative to the flowering stage (Burg and Burg, 1966; Min and Bartholomew, 1997; Van de Poel et al., 2009). In this study, some auxin-related genes, such as genes encoding auxin-induced protein, auxin-responsive protein, and auxin transporter-like protein, were isolated and were found to be differentially expressed (Additional Files 4, 5). These findings support those of the abovementioned previous studies.

The floral transition is a vital process for completion of the life cycles of higher plants. In *Arabidopsis*, previous molecular genetic studies have elucidated five genetically defined pathways that control flowering: the vernalization, photoperiod, autonomous, gibberellin, and age pathways (Srikanth and Schmid, 2011; Klepikova et al., 2015). These pathways converge to regulate the expression of several floral pathway integrators, including *FT, SOC1*/*AGL20, LFY*, and *FLC* (Boss et al., 2004; Parcy, 2005; Lei et al., 2013). They activate the downstream floral meristem identity genes *AP1, CAL*, and *FUL*, which in turn initiate the transition from the vegetative to the reproductive stage (Tani et al., 2009; Yu et al., 2013).

Vernalization, which can be defined as acceleration of flowering in plants, is promoted by a prolonged period of low temperatures (as typically experienced during the winter season), and it is considered to be one of the most specific primary factors for inducing and controlling reproductive development in some plant species (Tarkowská et al., 2012). In pineapple, ethylene is thought to be responsible for natural flowering at low temperatures (Botella et al., 2000). Nevertheless, there are few reports of genes related to low-temperature floral induction. In this study, an *LTI*-like (low temperature-induced) gene was found to be differentially expressed, exhibiting up-regulation in response to the two ethephon treatments. Recent molecular genetic approaches to understanding the regulatory pathway that promotes flowering in *Arabidopsis* have led to the conclusion that the main essential component of florigen is FLOWERING LOCUS T (FT) protein. *FT*-like genes are crucial regulators of flowering in angiosperms. In different plant species, *FT* homologs are involved in the earliest stages of flower development (Zeevaart, 2006). In this study, three *FT*-like genes were isolated from the plants exposed to the T2 treatment (the higher concentration of ethephon). In addition, one *FT*-like gene was found to be differentially expressed in the comparison of T2 with Ct. Similar results have been observed in a previous study (Lv et al., 2012b).

In wheat, barley and other monocotyledons species, the vernalization response is controlled by a MADS-box gene, *VERNALIZATION1* (*VRN1;* Kim et al., 2009; Alonso-Peral et al., 2011; Hemming and Trevaskis, 2011; Oliver et al., 2013; Yu et al., 2014). The *VRN1* gene of monocotyledons such as cereals plays a crucial and dual role in flowering; first, it induces expression of the cereal *FT* homolog through the vernalization pathway, and second, it acts as a floral meristem identity gene (Li and Dubcovsky, 2008). In this study, a *VRN1*-like gene was isolated from pineapple (a monocotyledon) and was found to be differentially expressed, exhibiting marked up-regulation in response to treatments T1 and T2 compared to Ct. These results indicate that the genes related to vernalization in pineapple are more closely related to vernalization genes in other monocotyledons than to those in dicotyledonous species and that ethephon induces expression of the *VRN1* gene.

In *Arabidopsis* and beet, the vernalization process promotes flowering by repressing the gene *FLOWERING LOCUS C* (*FLC*). It is known that the expression of this gene is increased by the action of the *FRIGIDA* (*FRI*) gene. The *FRI* and *FLC* genes, in contrast, act as constitutive floral inhibitors (Schmitz and Amasino, 2007; Crevillen and Dean, 2011; Pin et al., 2012; Yruela, 2015). In this study, three *FRI*-like genes were isolated from pineapple and were found to be differentially expressed, exhibiting down-regulation in response to the T1 and T2 ethephon treatments. These results indicate that stimulation with ethephon results in down-regulation of the expression of *FRI* genes.

The results of this study, including the observed up-regulation of the *LTI*-like and *VRN1*-like genes and down-regulation of the *FRI*-like genes, suggest that stimulation by ethephon may mimic the vernalization process induced by low temperatures in pineapple plants. Notably, further study is needed to elucidate the specific mechanism of ethephon-mediated stimulation rather than vernalization (low temperatures) in induction of flowering in pineapple.

After flowering initiation, floral meristems require the action of several FMI genes, such as *LFY, AP1, AG*, and *CAL*, the up-regulation of which controls floral organ identity and initiates the generation of floral meristems (Liu et al., 2008; Kaufmann et al., 2010; Han et al., 2014; Jung et al., 2014; Li D. M. et al., 2015). The *AP1, CAL*, and *FUL* genes act redundantly to control inflorescence architecture by influencing *LFY* and *TERMINAL FLOWER 1 (TFL1*) expression as well as their relative activity levels (Ferrándiz et al., 2000). Stamens and carpels are converted to petaloid organs, which is related to *AGAMOUS* (*AG*) gene expression (Akita et al., 2011; Sharifi et al., 2015). *AP1*-like genes specify floral meristem and organ identities (Shulga et al., 2011). *CAL* partially functions in the regulation of floral meristem identity and has a unique role in regulating cell differentiation during fruit development (Ferrándiz et al., 2000). These floral meristem identity genes cooperate to promote the transition from vegetative growth to reproductive growth (Zhang et al., 2013). In this study, three floral meristem identity genes, including *AP1*-like, *AG*-like, and *CAL*-like, were found to be differentially expressed in the comparison of T2 with Ct. Two of them, *AG*-like and *CAL*-like, were found to be differentially expressed in the comparison of T1 with Ct. The expression of these genes was up-regulated in response to stimulation by ethephon. These results suggested that stimulation by ethephon initiated the process of floral meristem identity and converted stamens and carpels to petaloid organs in pineapple by inducing specific genes. It also revealed the vital roles of the *AP1*-like, *AG*-like, and *CAL*-like genes in floral organ development and indicated that stimulation by ethephon induced the flowering of pineapple, perhaps by causing the increased expression of these flowering-related genes when it reaches the shoot apex (Turnbull et al., 1999). This information regarding flowering integration genes and FMI genes will facilitate more detailed studies of the mechanism of floral differentiation in pineapple.

In terms of the efficiencies of the two different concentrations of ethephon used for floral induction in this study, T1 and T2 induced floral transition in similar manners (Additional File 10) by up-regulating related genes. However, the T2 treatment, which contained the higher concentration of ethephon, solely up-regulated the *FT*-like and *AP1*-like genes, confirming that the efficiency of ethephon in floral induction in pineapple is determined by the ethephon concentration, the precise plant maturity level and climatic factors (Van de Poel et al., 2009).

The findings of this study provide a valuable resource for future pineapple genomic studies, and they can be applied in parallel studies of other closely related species. Notably, further studies are needed to verify the functions of the candidate genes identified in this study in floral induction in pineapple.

## Conclusion

In this study, we performed RNA-seq and gene expression profiling analyses of pineapple exposed to ethephon for stimulation of flowering to determine the genome-wide gene expression profile. An abundance of specific pineapple genes related to different biological functions were identified. Many DEGs were isolated and identified in the comparisons of the T1 and T2 ethephon treatments with Ct. The GO annotations of the DEGs revealed that the metabolic process and cellular process categories of the biological process ontology were predominant, as well as cell, cell part and organelle in the cellular component ontology, and catalytic activity and binding in the molecular function ontology. KEGG pathway analysis indicated that metabolic pathways, biosynthesis of secondary metabolites, and plant hormone signal transduction were enriched among the DEGs.

Thirteen DEGs were screened as candidate genes involved in ethephon-mediated stimulation and floral induction as well as floral meristem identity, including the *ERF*-like, *ETR*-like, *LTI*-like, *FT*-like, *VRN1*-like, *FRI*-like, *AP1*-like, *CAL*-like, and *AG*-like genes. The qPCR data obtained for these thirteen DEGs were in agreement with the corresponding RPKM values derived from RNA-seq, confirming the accuracy and credibility of the RNA-seq and gene expression profiling results. With regard to the comparison of T2 with Ct, two up-regulated DEGs, *FT*-like gene and *AP1*-like, were solely identified. Considering the screened candidate genes with increased expression, it appears that ethephon-mediated stimulation mimics the vernalization process of flowering induction in pineapple and that it promotes the expression of floral meristem identity genes involved in flower development.

The DEGs identified in this study are good candidates for functional analyses of pineapple flowering induced by stimulation with ethephon. The results of this study are valuable for future pineapple genomic studies, and they can be applied in parallel studies of other closely related species. However, further studies are needed to verify the functions of these candidate genes.

## Author contributions

CL carried out the experiments, performed the bioinformatics analyses, and wrote the manuscript. CF participated in the design and sampled the experimental materials.

### Conflict of interest statement

The authors declare that the research was conducted in the absence of any commercial or financial relationships that could be construed as a potential conflict of interest.

## Supplementary material

Additional File 1 **Primer sequences used in qPCR**.

Additional File 2 **COG classifications of unigenes**.

Additional File 3 **KEGG pathway assignments of unigenes**.

Additional File 4 **Differentially expressed genes (DEGs) in the comparison of T1 with Ct**.

Additional File 5 **DEGs in the comparison of T2 with Ct**.

Additional File 6 **DEGs in the comparison of T2 with T1**.

Additional File 7 **KEGG enrichment analysis results for DEGs in the comparison of T1 with Ct**.

Additional File 8 **KEGG enrichment analysis results for DEGs in the comparison of T2 with Ct**.

Additional File 9 **KEGG enrichment analysis results for DEGs in the comparison of T2 with T1**.

Additional File 10 **Flower bud of pineapple induced by T1 and T2**.
