# Supplementary figures and images for: De novo Transcriptome Assembly of Floral Buds of Pineapple and Identification of Differentially Expressed Genes in Response to Ethephon Induction
Source: Front Plant Sci. 2016 Feb 26;7:203. doi: 10.3389/fpls.2016.00203 (PMC4767906; doi:10.3389/fpls.2016.00203)

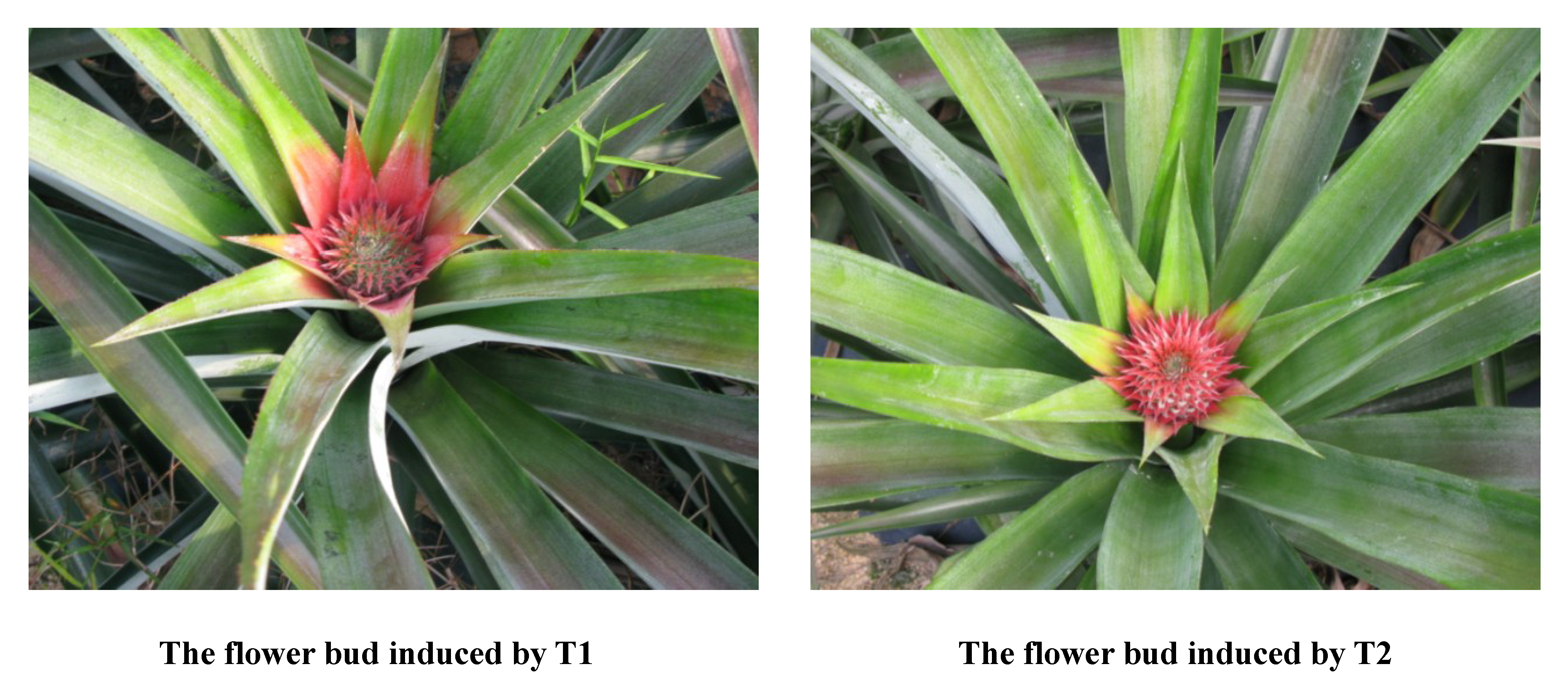

Supplement: Additional File 10 — Flower bud of pineapple induced by T1 and T2. [file Image1.tif]
